# Association between echinococcosis-specific health literacy and behavioural intention to prevent echinococcosis among herdsmen on the Tibet Plateau in China: a cross-sectional study

**DOI:** 10.1186/s12879-021-05775-8

**Published:** 2021-01-22

**Authors:** Jie Zhao, Yangzong Dawa, Kezhong A, Dejicuo, Lengbao, Zengyue Li, Wanli Chen, Jingya Wang, Yuxin Zhang, Dengbentai, Jiwei Wang, Lizheng Shi, Qingwu Jiang

**Affiliations:** 1 Key Laboratory of Public Health Safety of Ministry of Education, Key Laboratory of Health Technology Assessment of Ministry of Health, School of Public Health, Fudan University, 130 Dongan Road, Shanghai, 200032 China; 2 School of Philosophy, Fudan University, 220 Handan Road, Shanghai, 200433 China; 3 Qinghai provincial center for disease control and prevention, Xining, 810007 Qinghai Province China; 4 Center for Disease Control and Prevention of Gande County, Tibetan Autonomous Prefecture of Guoluo, 814100 Qinghai Province China; 5 Qinghai Provincial Institute for Endemic Disease Control and Prevention, Xining, 811602 Qinghai Province China; 6 School of Public Health and Tropical Medicine, Tulane University, New Orleans, LA 70112 USA

**Keywords:** Echinococcosis prevention, Health literacy, Behavioural intention

## Abstract

**Background:**

Echinococcosis is considered a neglected zoonotic disease and has been a major worldwide public health problem. Although it is known that health literacy is closely related to health behaviours and health outcomes, few studies have paid attention to echinococcosis related health literacy. This study aims to examine the association between echinococcosis-specific health literacy (ES-HL) and behavioural intention to prevent echinococcosis (BIPE) among herdsmen on the Tibet Plateauin in China.

**Methods:**

A cross-sectional study of 401 Tibetan herdsmen was conducted in Gande county of Qinghai Province, China. Participants were recruited from August to September 2018 and from February to March 2019. A self-developed questionnaire was used to measure demographic information, ES-HL and BIPE. Hierarchical regression analysis was done to identify the factors associated with BIPE.

**Results:**

In the hierarchical regression analysis, we entered age, sex, education level, marital state and family monthly income per capita into model 1 which explained a significant amount of variance in BIPE (Adjusted R^2^ change = 0.029, *P* = 0.006). Sex (β = − 0.125, *P* = 0.013) and family monthly income per capita (β = − 0.133, *P* = 0.009) were found to be associated with BIPE. Subsequently, the three factors of ES-HL were added to Model 1 to create Model 2. In Model 2, the two factors of ES-HL, perceived echinococcosis information support (β = 0.229, *P* < 0.001) and echinococcosis-specific self-management ability (β = 0.252, P < 0.001), were significantly associated with BIPE, while the information acquisition and evaluation ability factor (β =0.093, *P* = 0.089) was not found to be associated with BIPE. The model improved significantly when ES-HL was included (Model 2) explaining the 25.8% of variance of BIPE (Adjust R^2^ change =0.229, *P* < 0.001).

**Conclusions:**

ES-HL is an important predictor of whether individuals take preventive actions against echinococcosis. An ES-HL promotion action project should be developed targeting specific populations to enhance the prevention of echinococcosis.

## Background

Echinococcosis is a chronic zoonotic parasitic disease caused by infection of larval stages of cestodes of the genus Echinococcus [1]. Carnivores such as domestic dogs, wolves and foxes are the main source of infection, while herbivorous and omnivorous animals such as cattle and sheep act as intermediate hosts [2]. Alveolar echinococcosis (AE) caused and cystic echinococcosis (CE) are the two forms that pose a major public health threat to humans [3]. In 2015, the WHO estimated that echinococcosis caused nearly 20,000 deaths and resulted in more than 870,000 disability-adjusted life years globally each year [4].

Since the mid-1990s, the prevalence of echinococcosis in the Tibetan Plateau has been a major neglected public health problem, especially in the eastern and central regions of the Plateau [5]. Studies have shown that the burdens of CE and AE in Tibetan Plateau are higher than that in other endemic areas in the world [6, 7]. The human incidence of echinococcosis in some countries on the Tibetan Plateau can reach more than 50 cases per 100,000 people per year, with the prevalence exceeding 10% [8].

There are many social factors favouring the life cycle of the genus Echinococcus. In China, most Tibetan herdsmen families traditionally have close contact with their livestock and keep at least 1 dog. As a result of this tradition, dog manure and cow dung are commonly found scattered in their tents. Women collect these droppings by hand, dry them for fuel, and make fire with dung cakes when cooking. Poor hand washing habits and hand-kneaded food increase the chances of ingesting Echinococcus eggs. In addition, home slaughter and the feeding of dogs with raw offals favour the parasite’s life cycle. Furthermore, herders are often reluctant to harm animals, so they often refuse to deworm their dogs or vaccinate their lambs. These lifestyle factors which increase the risk of echinococcosis infection in herdsmen may be closely related to their lower health literacy (HL) [9].

The World Health Organization defines HL as ‘the cognitive and social skills which determine the motivation and ability of individuals to gain access to, understand, and use information in ways which promote and maintain good health’ [10]. HL has been considered to be an important goal of public health education strategies [11]. Low HL level is associated with difficulty in understanding disease knowledge, difficulty in adhering to health-promoting behaviours and underutilization of health services, resulting in increased morbidity and mortality [12]. Research has also shown that HL in patients with high-risk cardiovascular conditions is essential to self-care, especially in the most vulnerable populations [13]. Many studies have shown that diabetes patients with poor HL had difficulties adhering to all aspects of treatment regimens, because different knowledge and skills were required for unique aspects of the regimens [14, 15]. Echinococcosis-specific health literacy (ES-HL) refers to the abilities that enable the acquisition and processing of echinococcosis-related information and actively manage one’s health for the prevention and control of echinococcosis. We hypothesized that ES-HL is an important determinant of echinococcosis related health behaviours. Currently, there is a knowledge gap in this area with very little research considering the association between ES-HL and echinococcosis related health behaviours.

Behavioural intention refers to an individual’s judgment of the subjective probability of taking a particular behaviour. In some psychosocial behaviour models, behavioural intention has been regarded as a key determinant of the individual rational behaviour and has been employed extensively to understand social issues [16]. According to the theory of reasoned action, intention is the proximal predictor of behaviour [17]. The theory of planned behaviour also assumes that intention is the most important predictor of behaviour [18].

This study aims to examine the association between ES-HL and behavioural intention to prevent echinococcosis (BIPE) among herdsmen on the Tibet Plateau in China, so as to help inform and shape future interventions aimed at improving people’s echinococcosis related health behaviours.

## Methods

### Recruitment

**Fig. 1:**
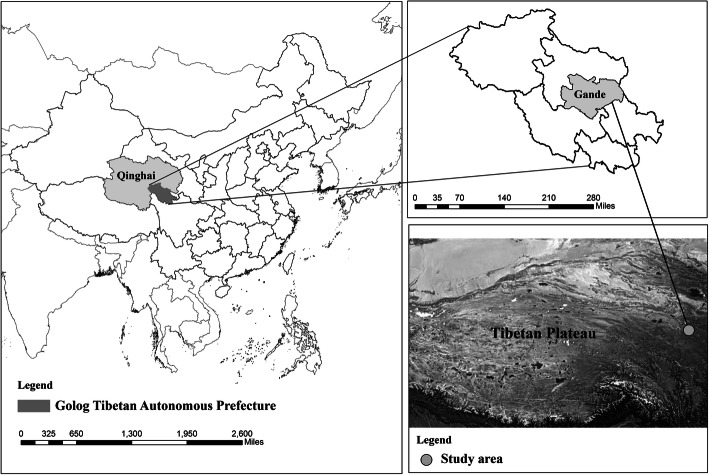
Location map showing the study area. Figure 1 was created by our own by using legitimate ArcGIS software (version 10.2, ESRI Inc., Redlands, CA, USA; https://desktop.arcgis.com/en/). Shapefiles was used to create vector maps of the border areas in ArcGIS software (We first downloaded GeoJSON-files at the free data platform: http://datav.aliyun.com/tools/atlas/ and then converted it to Shapefiles at the free website: https://mapshaper.org/). The Photo depicted in Figure 1 was obtained from Wikimedia Commons and the Photo was in the public domain (Wikimedia Commons is a free media repository: http://commons.wikimedia.org/wiki/Main_Page)

The cross-sectional study was conducted in Gande county, Golog Tibetan Autonomous Prefecture, Qinghai Province, China (Fig. 1). Gande county is located in the Southeastern part of Qinghai Province and in the hinterland of the Tibet Plateau. The area of Gande county is about 7046.2 km^2^ with an average elevation higher than 4300 m. Gande county is composed of 7 townships. There are 8579 households (including 7671 herdsman households), with a total population of 38,352 (including 32,523 herdsmen). The Tibetan population accounts for 98% of the total population. In addition, Tibetan Buddhism is by far the dominant religion in the area with a total of 11 Tibetan Buddhist monasteries in Gande county.

Based on the rule of ensuring 10 events per candidate predictor parameter (10 EPP) for prediction model [19], the sample size required for regression model analysis in this study is 130. For exploratory factor analysis, the sample size is required to be at least 10 times the number of items [20], and this study requires a sample size of 170. Therefore, the final sample size of this study should be no less than 170. The inclusion criteria of this study included: 1) Tibetan herdsmen; 2) 18–70 years old; 3) no serious concurrent diseases; 4) no serious mental illness or cognitive impairment; 5) no communication disorder. A convenient sampling method was adopted to recruit the participants from August to September 2018 and from February to March 2019. All 11 Tibetan Buddhist temples located in 7 townships in Gande county were selected as sampling sites for this study. Participants were recruited during 11 Buddhist activities which were held at the 11 temples. The number of herdsmen in each activity was 50 ~ 60, and a total of 640 herdsmen participated in the 11 activities. At the end of the activities, participants were introduced to the purpose and content of the survey by research team members. At the beginning of the introductions, 90 herdsmen left the temples mainly because they had no time to be surveyed. Five hundred fifty herdsmen listened to our introduction of the survey, but 149 of whom refused to participate. Some were not interested in the survey, while others were unwilling to wait for a long time and eager to rush home, because our team of 3–5 members could not survey all the herdsmen quickly. Finally, a total of 401 herdsmen participated in the survey and signed the informed consents. Questionnaires were conducted by an interviewer asking questions of a respondent in Tibetan in person. Each questionnaire took about 15 min to complete, with a $2 cash reward for completion.

### Measurement

#### Socio-demographic information

Socio-demographic information including age, sex, education level, marital state, family monthly income per capita was collected with self-report questionnaire.

#### Measurement of ES-HL

ES-HL was measured by a self-developed scale which was based on “The Health Literacy Questionnaire (HLQ) [21]” and “The European health literacy survey (HLS-EU) [22]”. The initial scale consists of 17 items designed to measure five ES-HL dimensions, namely “perceived echinococcosis information support (4items)”, “information acquisition ability (3items)”, “information evaluation ability (3items)”, “ability to actively communicate with health care providers (3items)”, and “echinococcosis-specific self-management ability (4items)”. Of these 17 items, eleven items were drawn from the HLQ, including three items from “ability to actively engage with healthcare providers” factor, three items from “ability to find good health information” factor, two items from “having sufficient information to manage my health” factor, one item from “appraisal of health information” factor, one item from “feeling understood and supported by healthcare providers” factor and one item from “actively managing my health” factor. Five items were drawn from the HLS-EU, including two items from “disease prevention-applying information on health” matrix, two items from “health promotion-applying information on health” matrix and one item from “disease prevention-appraising/judging information on health” matrix. Only one item was self-developed, which was “I can get advice from village cadres on echinococcosis prevention and treatment” in the dimension of “perceived echinococcosis information support”. Items drawn from existing instruments were slightly adapted to ensure their appropriateness for the echinococcosis-related situations. All items were rated on a 5-point Likert scale ranging from 1 = strongly disagree to 5 = strongly agree. The score of each item ranged from 1 to 5, with the overall score being the sum of all the item scores. To eliminate the impact of the number of items, scores for each dimension were obtained by dividing the total score of each dimension by its corresponding number of items. Therefore, the score of each dimension also ranged from 1 to 5.

#### Measurement of BIPE

BIPE was also measured by a questionnaire developed by our own. This questionnaire contains a total of 11 items, and all items were rated on a 5-point Likert scale ranging from 1 = very reluctant to 5 = very willing to. Participants were asked how strongly they were willing to take preventive action against echinococcosis by the questions such as “Are you willing to participate in the government-sponsored screening for echinococcosis?”, “Are you willing to deworm your dogs against echinococcosis?” or “Are you willing to bury the guts of a slaughtered animal rather than feed it to a dog?”. The score of each item ranges from 0 to 1, and the total score is the sum of all the item scores. In this study, the Cronbach’s α of the questionnaire was 0.882.

### Statistical analysis

Exploratory factor analysis (EFA) and confirmatory factor analysis (CFA) were used to test the structural validity of the ES-HL Scale. The 401 participants included in this study were randomized into two data sets (Set 1, *n* = 207; Set 2, *n* = 204) using the random case sampling feature of SPSS. EFA of the principal components with varimax rotation was performed on data set 1. An eigenvalue > 1 was used as a cut-off point to determine the applicability of the pre-designed factors of ES-HL. Items with factor loading > 0.5 and conceptual relevance were used as criteria for retaining in a factor. Items with factor loadings > 0.4 on two or more factors would be excluded. CFA was then performed on data set 2 using the method of maximum likelihood. Modification indices were used to guided adjustments to the relationships in the model and only the path of correlation was added between the residuals belonging to the same dimension. CFA was initially conducted on each of the factors with their corresponding items as the indicators. Moreover, CFA was conducted on all factors using their corresponding indicators ES-HL model.

Descriptive statistics were used to summarize the demographic characteristics of participants. Pearson correlation analyses was used to preliminarily analyze the relationship between study variables. Hierarchical multiple regression was used to explore the association between ES-HL and BIPE with age, sex, degree of education, marital status, and family monthly income per capita as control variables. The grouping of demographic variables is as follows: age (1 = “18–29”, 2 = “30–39”, 3 = “40–49”, 4 = “≥50”), sex (1 = “male”, 2 = “female”), marital status (1 = “married,” 2=“single, including unmarried, divorced, widowed and separated etc.”), education (1=“illiterate or primary school,” 2=“junior high school”, 3= “senior high school or above”), family monthly income per capita (1 = “< 1000”, 2 = “1000–1999”, 3 = “2000–3999”, 4 = “≥4000”). The demographic variables were set as dummy variables for correlation analysis and regression analysis. Tolerance and variance inflation factors were used to diagnose multicollinearity among independent variables.

In the present study, all statistical analyses were performed using the SPSS 22.0 and the AMOS 22.0. There was no missing value of demographic variables, and the number of missing values of ES-HL and BIPE variables were less than 5. The missing value was replaced by the continuous mean value method.

## Results

### Demographic characteristics

Table 1 summarizes the demographic characteristics of the respondents. In this study, 401 Tibetan pastoral residents were recruited. The mean age of the respondents is 38.61 ± 13.31 (mean ± SD), and 49.1% are female. The majority (74.6%) have just finished primary school or are illiterate, and 44.6% have a monthly income < 1000 CNY (≈142USD). More than 80% of respondents are married.

**Table 1:** Demographic characteristics of the respondents

Characteristic	Number (%)
**Sex**	
Male	197 (49.1)
Female	204 (50.9)
**Age**	
< 29	110 (27.4)
30–39	120 (29.9)
40–49	88 (21.9)
≥ 50	83 (20.7)
**Degree of education**	
Illiterate or primary school	299 (74.6)
Junior high school	38 (9.5)
Senior high school or above	64 (16.0)
**Marital status**	
Married	326 (81.3)
Others (unmarried, divorced, widowed)	75 (18.7)
**Family monthly income per capita**	
< 1000	179 (44.6)
1000–1999	112 (27.9)
2000–3999	75 (18.7)
≥ 4000	35 (8.7)

Table legend: Table 1 summarizes the demographic characteristics of the respondents

### Exploratory factor analysis

Table 2 presents the factor loadings based on EFA, Cronbach’s α, score mean and standard deviation for these factors. The results of KMO (kaiser-meyer-olkin) measurement (KMO value = 0.922) and Bartlett’s sphericity test (*P* < 0.001) confirmed the adequacy for the EFA. EFA of the initial scale yielded three factors that accounted for 69.51% of total variance. Two items of the initial scale with factor loadings > 0.4 on two or more factors were deleted. After analyzing and categorizing the meanings of the factors and the items they contain, the three factors are named for perceived echinococcosis information support, information acquisition and evaluation ability and echinococcosis-specific self-management ability. Perceived echinococcosis information support factor (4 items) contains the same items as the first dimension of the initial scale. The factor refers to the ability to obtain information support on echinococcosis control from various channels. Information acquisition and evaluation ability factor (5 items) mainly merges the second and the third dimensions of the initial scale. The factor refers to the ability to find information about echinococcosis independently and to make independent judgment and evaluation. Echinococcosis-specific self-management ability factor (6 items) mainly merges the fourth and the fifth dimensions of the initial scale. The factor refers to the ability to participant in health promotion activities for echinococcosis prevention.

**Table 2:** Results of exploratory factor analysis of ES-HL

	Factor loading	Reliability (Cronbach’s α)	Mean ± SD
**Perceived echinococcosis information support**		0.880	2.964 ± 0.968
Be able to get advice on echinococcosis control from health professionals	0.664		
Be able to get advice on echinococcosis control from family members	0.756		
Be able to get advice on echinococcosis control from friends	0.787		
Be able to get advice on echinococcosis control from village officials	0.791		
**Information acquisition and evaluation ability**		0.914	2.402 ± 0.954
Be able to find information on echinococcosis control from several different sources	0.814		
Be able to find echinococcosis-related information independently	0.825		
Be able to analysis/evaluation echinococcosis-related information	0.861		
Have enough information to help dealing with the problems about echinococcosis	0.848		
Be sure that knowledge about echinococcosis has been mastered	0.811		
Be able to discuss echinococcosis with health care provider	0.590		
**Echinococcosis-specific self-management ability**		0.874	3.163 ± 0.848
Be able to give a good description of daily behaviour	0.722		
Be able to obtaine information on echinococcosis control by communication with medical and health personnel	0.739		
Be able to prevent echinococcosis through active behaviour management	0.757		
Participate in screening for echinococcosis actively	0.836		
Attend lectures and activities on echinococcosis control actively	0.750		

Table legend: Table 2 presents the factor loadings based on EFA, Cronbach’s α, score mean and standard deviation for these factors

### Confirmatory factor analysis

Table 3 summarizes the modified fit statistics of CFA of ES-HL model. CFA was conducted respectively on perceived echinococcosis information support factor with four indicators, information acquisition and evaluation ability factor with six indicators and echinococcosis-specific self-management ability factor with five indicators. The fit indices of the three models suggest that each of the three factors is appropriate. To confirm the factor structure, the three-factor-model of ES-HL was tested using CFA. The fit indices show that the model fits the data well.

**Table 3:** Fit statistics (modified) of CFA of ES-HL model (*n* = 204)

Model	χ^2^ (df)	GFI	AGFI	TLI	CFI	RMSEA	SRMR
Three-factor model of ES-HL	174.719 (80)	0.900	0.850	0.945	0.958	0.075	0.067
Single-factor model of perceived echinococcosis information support	1.184 (1)	0.997	0.971	0.998	1.000	0.030	0.007
Single-factor model of information acquisition and evaluation ability	5.182 (7)	0.976	0.937	0.984	0.991	0.070	0.012
Single-factor model of echinococcosis-specific self-management ability	9.244 (3)	0.982	0.910	0.964	0.989	0.101	0.022

Table legend: *GFI* Goodness-of-fit index, *AGFI* Adjusted GFI, *CFI* Comparative fit index, *TLI* Tucker Lewis index, *RMSEA* Root mean square error of approximation, *SRMR* Standardized root mean square residual

### Preliminary correlation analyses

Table 4 summarizes the correlation among the demographic variables (sex, age, education level, marital status, family monthly income per capita), ES-HL and BIPE. The table indicates sex, family monthly income per capita, and ES-HL as significantly correlated with the BIPE. Results from the collinearity statistics test also show that all variables have a tolerance greater than 0.5 and the variance inflation factor is less than 2, which means that there is no multicollinearity among independent variables.

**Table 4:** Correlation among demographic variables, ES-HL and BIPE

Characteristic	1	2	3	4	5	6	7	8	9
1. Sex	–								
2. Age	−0.102*	–							
3. Degree of education	0.064	−0.383***	–						
4. Marital status	0.126*	−0.357***	0.384***	–					
5. Family monthly income per capita	−0.004	− 0.013	0.159**	− 0.017	–				
6. Perceived echinococcosis information support	−0.09	−0.141**	0.053	0.015	−0.031	–			
7. Information acquisition and evaluation ability	−0.023	−0.243***	0.269***	0.044	0.081	0.527**	–		
8. Echinococcosis-specific self-management ability	−0.131**	0.064	−0.032	−0.065	− 0.04	0.646**	0.371***	–	
9. BIPE	−0.137**	0.042	−0.026	−0.092	− 0.127*	0.439***	0.283**	0.454**	–

Table legend: Table 4 summarizes the correlation among the demographic variables (sex, age, education level, marital status, family monthly income per capita), three factors of ES-HL and BIPE. Demographic variables were all categorical variables, which were set as dummy variables for correlation analysis. * *P* < 0.05, ** *P* < 0.01,*** *P* < 0.001

### Hierarchical multiple regression analysis

Table 5 reports the unstandardized (B) and standardized (β) regression coefficients for model 1 and model 2. For model 1, sex, age, education, marital status, and income were entered into the model. Sex (β = − 0.125, *P* = 0.013) and family monthly income per capita (β = − 0.133, *P* = 0.009) explained a significant amount of variance of BIPE (Adjust R^2^ change =0.029, *P* = 0.006). The model improved significantly when ES-HL was included (Model 2) explaining 25.8% of variance of BIPE (Adjust R^2^ change =0.229, *P* < 0.001). In model 2, sex (β = − 0.064, *P* = 0.152) was no longer significantly related to BIPE, while family monthly income per capita (β = − 0.119, *P* = 0.008) was still significantly related to BIPE. Perceived echinococcosis information support (β = 0.229, P < 0.001) and echinococcosis-specific self-management ability (β = 0.252, P < 0.001) were significant predictors of BIPE, while the dimension of information acquisition and evaluation ability (β = 0.093, *P* = 0.089) was not significantly related to BIPE.

**Table 5:** Summary of hierarchical regression analysis for variables predicting the BIPE

Variable	*B*	SE *B*	β	△Adjust R^2^	Total adjust R^2^
**Model 1**				0.029	0.029
Age	0.082	0.342	0.013		
Sex	−1.676	0.673	−0.125*		
Degree of education	0.390	0.511	0.044		
Marital status	−1.533	0.958	−0.090		
Family monthly income per capita	−0.901	0.343	−0.133**		
**Model 2**				0.229	0.258
Age	0.370	0.310	0.060		
Sex	−0.851	0.593	−0.064		
Degree of education	0.083	0.460	0.009		
Marital status	−1.028	0.843	−0.060		
Family monthly income per capita	−0.808	0.301	−0.119**		
Perceived echinococcosis information support	1.581	0.437	0.229^***^		
Information acquisition and evaluation ability	0.649	0.380	0.093		
Echinococcosis-specific self-management ability	1.998	0.463	0.252^***^		

Table legend: Table 5 reports the unstandardized (B) and standardized (β) regression coefficients for model 1 and model 2. Demographic variables were all categorical variables, which were set as dummy variables for regression analysis.^*^ *P* < 0.05, ^**^ *P* < 0.01,^***^ *P* < 0.001

## Discussion

This study shows that the two dimensions of ES-HL, perceived echinococcosis information support and echinococcosis-specific self-management ability, were significantly associated with BIPE, while the information acquisition and evaluation ability dimension was not found to be associated with BIPE. In addition, sex and income level were also associated with BIPE.

In this study, perceived echinococcosis information support factor involves the use of cognitive and interactive skills in a social environment. It is associated with the extent to which people participate in health-related community activities and exchange health information. Studies have shown that social support is crucial to the spread of health information among populations and has an important impact on individuals’ health behaviours [23, 24]. This study shows that when herdsmen have more perception of echinococcosis information support from the village cadres, the medical staff or family members, their BIPE will increase. This may be because more social information support means more access to information, then the herdsmen will have a better understanding of echinococcosis or are more likely to be motivated by others to take action to prevent echinococcosis. Consistent with our research, in the case of oral health-promoting behaviour, it was indicated that the health literacy domain “Feeling understood and supported by healthcare provider” in HLQ was associated with oral health-promoting behaviour, including the frequency of brushing teeth and the use of interdental floss [25].

The researchers believed that the definition of self-management includes at least two components: self-supervision and decision-making [26]. In this study, echinococcosis-specific self-management skills include the abilities to conduct self-assessment, participate in echinococcosis prevention services and make decisions to modify their own behaviour. At present, self-management ability has been widely concerned in the management of chronic diseases [27–29]. Many studies have also shown that self-management interventions have benefits for the prevention of infectious diseases, such as AIDS [30], tuberculosis [31], hepatitis C [32] and filariasis [33]. Furthermore, it has been found that health literacy domain “active managing of one’s health” in HLQ was closely related to the health risk behaviours of Danish adults, including physically inactive, unhealthy diet and smoking [34], as well as the oral health-promoting behaviours of Slovak adults [25]. It was also indicated that “active managing of one’s health” domain was significantly associated with the lifestyle risk factors including smoking and being more sedentary among Geelong Osteoporosis [35]. The result of our study that echinococcosis-specific self-management ability was a key predictor of individual BIPE is consistent with the above studies on the relationship between health behaviours and self-management ability in health literacy domain.

In this study, information acquisition and evaluation ability factor was not found to be associated with BIPE. This may be explained by the fact that most respondents recruited in this study have just finished primary school or were illiterate. Information acquisition and evaluation ability of most participants are generally at a very lower level. Therefore, it may be not a sensitive predictor of BIPE. Furthermore, most herdsmen live in resource-limited and deprived communities, and their understanding of echinococcosis rely heavily on their social networks. Therefore, echinococcosis information channels and external echinococcosis information support may be more important for echinococcosis prevention than their own information acquisition and evaluation ability.

In addition, this study shows that sex is an influencing factor of the BIPE. Compared with males, the BIPE for females is lower in this study. There is also a study that showed the gap of echinococcosis-related knowledge between men and women [36]. For example, most men were aware of the link between infected offal and dog infection, but few women were [36]. A health information intervention study on echinococcosis [37] also showed that the intervention had different outcomes among men and women because of the educative role and norms at the household level. Our study also shows that the BIPE in high-income earners was lower than that in low-income earners. This may be explained by the fact that high-income earners have more cattle and sheep, and they have to spend more time and energy on herd care, so they are more likely to ignore the prevention of echinococcosis.

Although many effective health literacy assessment tools have been developed [38], there is still a lack of research on ES-HL assessment tools. The current study develops a three-factor ES-HL scale with good reliability and structure validity. The three factors are significantly correlated with BIPE and could be regarded as important targets to guide echinococcosis related health education intervention campaigns.

This study has several limitations. First, this study is a cross-sectional survey, so the causal relationship between research variables cannot be fully inferred. Second, the measurement tools in this study are self-developed questionnaires which have certain measurement bias. The reliability and validity of the tools still need to be verified in future studies. Third, the convenience sampling technique was applied to recruit participants, so the sample may not be fully representative and limits the statistical inference. In Gande county, a vast area, many herdsmen live scattered in remote mountainous areas and have strong mobility, and many of whom do not use mobile phones because of the bad reception. It is difficult to reach participants through other sampling methods. Giving the fact that almost everyone in Gande county is a Follower of Tibetan Buddhism and will participate in the Buddhist activities regularly, the temples are the most suitable sampling sites where herdsmen can be widely reached. Fourth, we could only recruited the herdsmen from one Buddhist activity at each temple, so many herdsmen who took part in the Buddhist activities in non-survey period were out of our reach. Fifth, the response rate is low, and the study may suffer from a non-response bias. In future similar studies, considerations should be made to train medical personnel or Buddhist monks to investigate the herders to improve their compliance with the survey, or increase the reward for completing the survey. Timing of the investigation is also important, such as in the early afternoon, to prevent herders from rushing home. Finally, we cannot completely exclude the fact that herdsmen may have previously received echinococcosis related health education interventions, which could impact on the knowledge, attitude [39–41] and subjective norms [42] related to BIPE of herdsmen and was an important confounding factor.

## Conclusions

ES-HL is an important predictor of whether individuals take preventive actions against echinococcosis. An ES-HL promotion action project should be developed targeting specific populations to enhance the prevention of echinococcosis.

## Data Availability

The datasets used and/or analysed during the current study are available from the corresponding author on reasonable request.

## Abbreviations

ES-HL: Echinococcosis-Specific Health Literacy
BIPE: Behavioural Intention to Prevent Echinococcosis
AE: Alveolar Echinococcosis
CE: Cystic Echinococcosis
HL: Health Literacy
HLQ: The Health Literacy Questionnaire
HLS-EU: The European Health Literacy Survey
EFA: Explore Factor Analysis
KMO: Kaiser-Meyer-Olkin
